# Comparison of Brief Cognitive Tests and CSF Biomarkers in Predicting Alzheimer’s Disease in Mild Cognitive Impairment: Six-Year Follow-Up Study

**DOI:** 10.1371/journal.pone.0038639

**Published:** 2012-06-22

**Authors:** Sebastian Palmqvist, Joakim Hertze, Lennart Minthon, Carina Wattmo, Henrik Zetterberg, Kaj Blennow, Elisabet Londos, Oskar Hansson

**Affiliations:** 1 Clinical Memory Research Unit, Department of Clinical Sciences, Lund University, Malmö, Sweden; 2 Department of Psychiatry and Neurochemistry, Institute of Neuroscience and Physiology, The Sahlgrenska Academy at the University of Gothenburg, Mölndal, Sweden; McGill University/Douglas Mental Health Univ. Institute, Canada

## Abstract

**Introduction:**

Early identification of Alzheimer’s disease (AD) is needed both for clinical trials and in clinical practice. In this study, we compared brief cognitive tests and cerebrospinal fluid (CSF) biomarkers in predicting conversion from mild cognitive impairment (MCI) to AD.

**Methods:**

At a memory clinic, 133 patients with MCI were followed until development of dementia or until they had been stable over a mean period of 5.9 years (range 3.2–8.8 years). The Mini-Mental State Examination (MMSE), the clock drawing test, total tau, tau phosphorylated at Thr_181_ (P-tau) and amyloid-β_1–42_ (Aβ_42_) were assessed at baseline.

**Results:**

During clinical follow-up, 47% remained cognitively stable and 53% developed dementia, with an incidence of 13.8%/year. In the group that developed dementia the prevalence of AD was 73.2%, vascular dementia 14.1%, dementia with Lewy bodies (DLB) 5.6%, progressive supranuclear palsy (PSP) 4.2%, semantic dementia 1.4% and dementia due to brain tumour 1.4%. When predicting subsequent development of AD among patients with MCI, the cognitive tests classified 81% of the cases correctly (AUC, 0.85; 95% CI, 0.77–0.90) and CSF biomarkers 83% (AUC, 0.89; 95% CI, 0.82–0.94). The combination of cognitive tests and CSF (AUC, 0.93; 95% CI 0.87 to 0.96) was significantly better than the cognitive tests (p = 0.01) and the CSF biomarkers (p = 0.04) alone when predicting AD.

**Conclusions:**

The MMSE and the clock drawing test were as accurate as CSF biomarkers in predicting future development of AD in patients with MCI. Combining both instruments provided significantly greater accuracy than cognitive tests or CSF biomarkers alone in predicting AD.

## Introduction

The early identification of Alzheimer’s disease (AD) is becoming increasingly important so that the correct care and follow-up can be initiated [1], [2]. Many on-going studies address disease-modifying treatments for AD, and in the future it will probably be important to identify AD patients very early in order to commence these treatments in time [3]. AD is generally preceded by an incipient preclinical phase [4], which progresses to mild cognitive impairment (MCI) [5] and finally to dementia [6]. MCI is not only caused by incipient dementia, but has many different causes and varying patterns of progression. To identify patients with AD at an early stage, it is therefore important to identify specifically those MCI patients who will later convert to AD.

A most successful biomarker-based method of predicting the conversion from MCI to AD has been the analysis of cerebrospinal fluid (CSF) and in particular total tau (tau), tau phosphorylated at Thr_181_ (P-tau) and the 42-amino-acid isoform of amyloid-β_1–42_ (Aβ_42_). These analyses have provided classification accuracies of around 80% or even more. [7]–[9] Unfortunately, these analyses are not available everywhere, and are unlikely to become a standard procedure because of the increasing prevalence of dementia, especially in developing countries, and the fact that the majority of patients must be evaluated in primary care.

Another way to predict AD is by administering brief cognitive tests. The most commonly used cognitive tests for dementia screening are the Mini-Mental State Examination (MMSE) and the clock drawing test [10]. Several studies have examined their ability to predict AD, but with highly varying results ranging from zero predictive ability to values similar to those of CSF [11]–[16]. One way to establish their predictive ability is compare their performance with well-validated CSF biomarker. It would also be useful if the brief cognitive tests were as accurate as CSF biomarkers in predicting AD, because cognitive tests are available everywhere, can be administered quickly, are very cheap and well tolerated by patients. In addition to comparing the MMSE and clock drawing test with CSF, it would be useful to know whether they complement one another, providing additional diagnostic value, especially in clinics that use both CSF analysis and cognitive tests.

To our knowledge, no previous article has compared the clock drawing test and the MMSE with CSF biomarkers for the prediction of AD. Only two different studies have ever reported on any kind of cognitive test and CSF biomarkers in the same MCI study; a Swedish MCI study and the Alzheimer’s Disease Neuroimaging Initiative (ADNI) [11], [17]–[19]. The Swedish MCI paper did not have the aim of comparing the methods and the ADNI papers only followed the patients for about two years and did not examine brief cognitive tests suitable for primary care.

The primary aim of this study was to compare the abilities of the MMSE and the clock drawing test with CSF biomarkers to predict AD among MCI patients. Our secondary aim was to investigate the additional diagnostic value achieved by combining the cognitive tests with CSF biomarkers.

## Materials and Methods

### The MCI Population

This study was conducted at the Memory Clinic of Skåne University Hospital in Malmö, Sweden. The MCI cohort consisted of patients referred to the clinic between October 2000 and January 2006. Most patients were referred from primary care units, but some referrals came from other clinics at the hospital. The specific procedures of this cohort have been described in greater detail elsewhere [20]. At the initial visit, all patients were assessed by physicians experienced in dementia disorders, and underwent thorough physical, psychiatric and neurological examinations, as well as an interview that focused on their cognitive symptoms and ADL function. The patients also underwent a computed tomography or magnetic resonance imaging of the brain, lumbar puncture, cognitive tests and routine blood analysis, including assessment of their apolipoprotein E (APOE) genotype. The MCI criteria proposed by Petersen and colleagues were applied [21], i.e. 1) memory complaints of the patient, but preferable also acknowledged by an informant; 2) objective memory impairment in relation to age and education, assessed by the physician; 3) a relatively preserved general cognition based on the physicians structural interview and a MMSE score of at least 24 points; 4) intact or very slightly impaired ADL; and 5) not fulfilling the DSM-IIIR criteria for dementia [22].

In the present study, 133 patients with MCI at baseline were included. The patients were followed over time with repeated clinical visits, until development of either a specific type of dementia or until they had been cognitively stable (stable MCI) for 5.9 years (range 3.2–8.8 years). AD was diagnosed as probable AD according to NINCDS-ADRDA [23], vascular dementia (VaD; either probable VaD according to NINDS-AIREN [24] or subcortical VaD according to Erkinjuntti and colleagues [25]) or dementia with Lewy bodies (DLB) according to the McKeith criteria [26]. A consensus group of three study physicians experienced in dementia disorders (OH, JH and LM) later determined all diagnoses. The physicians were blinded to the CSF and cognitive test data collected on the initial visit.

The Regional Ethics Committee in Lund, Sweden, approved the study design and the consent. All patients gave their written informed consent. The data were analyzed anonymously.

### The MMSE and the Clock Drawing Test

The maximum score of the MMSE is 30 and the test consists of the following parts: time and place orientation (10 points), word registration (3 points), attention (5 points), delayed word recall (3 points), various verbal tasks (8 points) and visuo-construction (1 point) [27]. The attention part was administered using the serial 7s task. Spelling backwards was only used if the patient could not perform serial 7s. Apart from the total MMSE score, the combined score of orientation and delayed word recall was also used as a variable, hereafter referred to as MMSE (orientation & recall). These two parts have previously been shown to be the best MMSE predictors of future AD [15], [16], [28].

The clock drawing test was administered on a blank piece of paper and the patients were instructed to draw the face of a clock with all the numbers on it and set the time to 10 after 11. The test was scored according to Shulman et al. [29], since this scoring method has been better at predicting AD compared to other scoring methods [11]. Five points were given for a perfect clock and 0–4 points depending on the severity of the errors.

### CSF Analysis

CSF was collected at baseline in polypropylene tubes and gently mixed to avoid gradient effects. All samples were centrifuged within 30 minutes at +4°C at 2000 g for 10 min to remove cells and debris. Samples were stored in aliquots at −80°C pending biochemical analysis. The procedure used and the analysis of the CSF followed the Alzheimer’s Association Flow Chart for lumbar puncture [30]. The Luminex xMAP technology was used to determine the levels of tau, Aβ_42_ and P-tau [31]. In addition to tau, Aβ_42_ and P-tau, the ratio of Aβ_42_/tau was tested as a separate variable in the logistic regression models since it previously has shown high predictive accuracy in this cohort [20]. Lumbar puncture was only conducted at the initial visit.

### Statistical Analysis

The categorical variables sex and presence of the APOE ε4 allele were analysed using the χ^2^ test. All non-categorical variables were compared using the Kruskal-Wallis one-way analysis of variance. If this test was significant, Mann-Whitney U test was performed. Sensitivity and specificity were calculated using the receiver operating characteristic (ROC) curve analysis. ROC curve analysis was first performed on single CSF and cognitive test variables. The cut-off which produced the highest Youden index (sensitivity + specificity –1) was chosen. The method of DeLong et al. (implemented in MedCalc) was used to compare the ROC curves of single variables and the logistic regression models [32].

The ability of the CSF biomarkers and cognitive tests to predict dementia and AD was also examined with logistic regression analysis using the backward likelihood ratio (LR) method. Sex and age were adjusted for in the regression models. Before entering the clock drawing data into the regression models this variable was dichotomized because of the small number of patients at the more impaired levels. The original cut-off by Shulman of <4 points was used [29], [33].

The variables were screened for multicollinearity using the Spearman correlation, because multicollinearity can cause unstable models. There were strong correlations between the MMSE and MMSE (orientation & recall) (r = 0.89; p<0.001) and between tau and Aβ_42_/tau (r −0.88; p<0.001). The models that included tau and MMSE (orientation & recall) produced better accuracies than the models with Aβ_42_/tau and the MMSE. Therefore, the latter two variables were removed to reduce collinearity. Aβ_42_ was entered separately both as a continuous variable and as a dichotomised variables (cut-off according to the highest Youden index, which was <208). The variable that classified most patients correctly was used.

To compare the models that included the CSF variables with the models that included the cognitive test variables, the probabilities of each model were saved as a new variable (a value between 0 and 1 for each individual). These variables were then used to plot ROC curves and to compare the areas under the curves (AUCs). The comparison of AUCs has previously been used to compare logistic regression models [12].

A p value of <0.05 was considered statistically significant (hereafter referred to as “significant”). The ROC analyses, including the comparisons of the AUCs, were performed with MedCalc version 11.5.1 (MedCalc Software, MariaKerke, Belgium). All other analyses were performed with SPSS software, version 19.0.0 (SPSS Inc., Chicago, IL).

## Results

Of the 133 patients with MCI at baseline, 53.4% (71 patients) developed dementia and 46.6% (62 patients) remained stable during the mean follow-up period of 5.9 years (range 3.2–8.8 years). The dementia incidence was 13.8% per year and 10.1% for AD specifically. Among those who developed dementia, the prevalence of AD was 73.2% (52 patients), VaD 14.1% (10 patients), DLB 5.6% (4 patients), progressive supranuclear palsy (PSP) 4.2% (3 patients), semantic dementia 1.4% (1 patient) and dementia due to brain tumour (according to DSM-IV [34]) 1.4% (1 patient). The patients with VaD, DLB, PSP, semantic dementia and dementia due to brain tumour, were grouped as “MCI-other dementias”. The demographics of the different groups are shown in table 1. All CSF and cognitive test variables differed significantly between patients with MCI who subsequently developed AD (MCI-AD) and cognitively stable patients with MCI (stable MCI). Patients with MCI-other dementias had lower MMSE score, but not MMSE (orientation & recall) score, compared to stable MCI. Sex, MMSE, MMSE (orientation & recall), tau, Aβ_42_, P-tau and Aβ_42_/tau differed significantly between MCI-AD and MCI-other dementias (Table 1).

**Table 1:** Baseline variables.

Variable	Stable MCI (N = 62)	MCI-AD (N = 52)	MCI-Otherdementias (N = 19)	Significant difference
Age, mean (range)	69.8 (55–85)	75.3 (55–87)	71.2 (59–83)	MCI-AD > Stable MCI*** MCI-AD > MCI-Other*
Sex, female	55%	70%	42%	MCI-AD > MCI-Other*
APOEε4, ≥ one allele	45%	76%	63%	MCI-AD > Stable MCI***
MMSE, mean ± SD	28.1±1.2	26.1±1.5	27.1±2.0	Stable MCI > MCI-AD*** Stable MCI > MCI-Other*
MMSE (O & R), mean ± SD	11.4±1.1	9.6±1.4	10.9±1.3	Stable MCI > MCI-AD*** MCI-Other > MCI-AD***
Clock drawing, mean ± SD	4.7±0.6	4.0±1.0	4.3±0.9	Stable MCI > MCI-AD***
Tau, mean ± SD	78.1±44.3	141.5±71.2	78.8±39.5	MCI-AD > Stable MCI*** MCI-AD > MCI-Other**
Aβ_42_, mean ± SD	244.9±63.7	155.2±57.9	214.7±64.8	MCI-Other > MCI-AD*** Stable MCI > MCI-AD***
P-tau, mean ± SD	30.0±16.6	49.0±22.5	26.0±11.5	MCI-AD > Stable MCI*** MCI-AD > MCI-Other***
Aβ_42_/Tau, mean ± SD	4.0±1.9	1.5±1.2	3.2±1.5	MCI-Other > MCI-AD*** Stable MCI > MCI-AD***

* p<0.05;

** p<0.01;

*** p<0.001.

AQT  =  A Quick Test of Cognitive Speed; MCI-AD  =  MCI patients who progress to AD; MCI-Other dementias  =  MCI patients who progress to other dementias than AD; MMSE (O & R)  =  the orientation and delayed word recall parts of the MMSE; SD  =  standard deviation.

MMSE, MMSE (O&R), Clock drawing, Aβ_42_ and Aβ_42_/Tau: A lower value is pathological.

AQT, Tau and P-tau: A higher value is pathological.

There were significant differences among the groups for all variables (Kruskal-Wallis).

### Prediction of AD with ROC Curve Analysis

The sensitivity and specificity of each CSF and cognitive test variable are shown in Table 2. The variable with the best AUC to differentiate MCI-AD from stable MCI and MCI-other dementias was Aβ_42_ (AUC 0.84, 95% CI 0.77–0.90), followed by MMSE (orientation & recall) (AUC 0.82, 95% CI 0.74–0.88). The MMSE, MMSE (orientation & recall) and all CSF variables had a significantly better AUC than the clock drawing test. Otherwise there were no significant differences between the variables.

**Table 2:** Comparison of single variables for predicting follow-up diagnoses (ROC curve analysis).

	MCI-AD (N = 52) compared with Stable MCI and MCI-other dementias (N = 81)			
*Variable*	*AUC (95% CI)*	*Cut-off*	*Sensitivity, % (95% CI)*	*Specificity, % (95% CI)*
MMSE	0.79 (0.71–0.86)*	<27 points	62 (47–75)	84 (74–91)
Clock drawing	0.67 (0.58–0.75)	<4 points	44 (31–59)	86 (77–93)
MMSE (O & R)	0.82 (0.74–0.88)**	<10 points	54 (40–68)	94 (86–98)
Tau	0.81 (0.73–0.87) *	>87 pg/ml	80 (66–90)	72 (61–82)
Aβ_42_	0.84 (0.77–0.90)**	<208 pg/ml	90 (79–97)	69 (58–79)
P-tau	0.79 (0.72–0.86)*	>39 pg/ml	67 (53–80)	86 (77–93)

* p<0.05; ** p<0.01; compared with AUC of clock drawing.

The cut-offs were chosen to yield the highest Youden index.

Clock drawing was scored according to Shulman {Shulman, 2000 #36}.

CI =  Confidence interval, MMSE (O & R)  =  The orientation and delayed word recall parts of the MMSE.

The best combination of CSF biomarkers was the tau/Aβ42 ratio. At <1.6 the sensitivity was 74% and the specificity 92% (AUC 0.88, 95% CI 0.81–0.93). A combined MMSE (orientation & recall) score and clock drawing score of less than 15 points produced a sensitivity of 71% and a specificity of 84% (AUC 0.84, 95% CI 0.77–0.90). These two combinations did not differ significantly (p = 0.44).

### Prediction of AD with Logistic Regression Analysis

**Figure 1:**
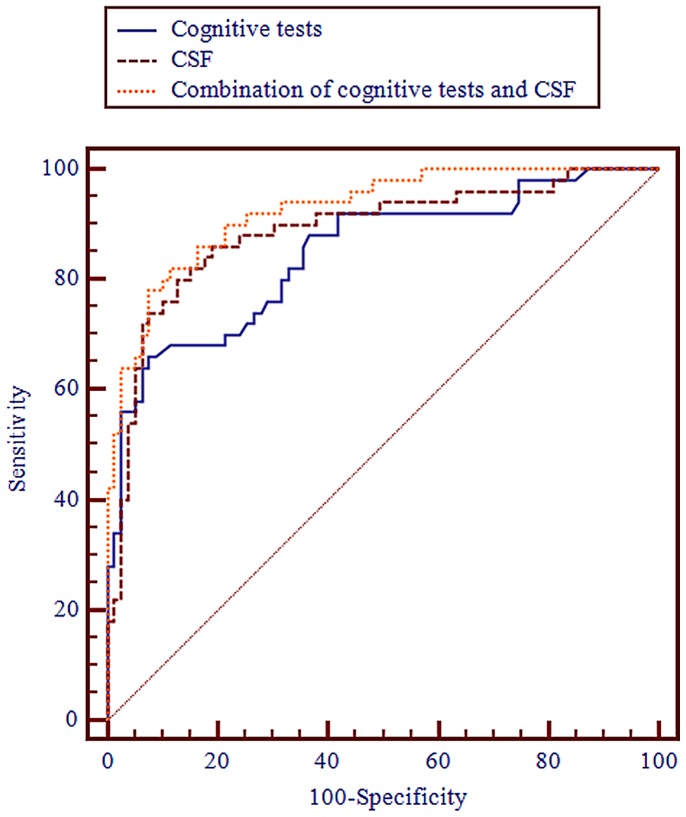
AUCs for MCI-AD compared with stable MCI and MCI-other dementias. The AUCs were derived from the logistic regression models (Table 3). The AUC from the combined model with both cognitive tests and CSF biomarkers was significantly better than that of the CSF model (p = 0.04) and the cognitive test model (p = 0.01). MMSE (O & R)  =  the orientation and delayed word recall parts of the MMSE.

When predicting AD compared with stable MCI and MCI-other dementias, the MMSE (orientation & recall), clock drawing (dichotomised at <4 points) and age classified 81% of the cases correctly (AUC 0.85, 95% CI 0.77–0.90). Aβ_42_, tau and age classified 83% correctly (AUC 0.89, 95% CI 0.82–0.94). No significant difference was found between the AUCs obtained using cognitive tests or CSF biomarkers (p = 0.36, Table 3). In the combined model, MMSE (orientation & recall), Aβ_42_ (dichotomised at <208), tau and clock drawing (dichotomised at <4 points) classified 85% of the patients correctly (AUC 0.93, 95% CI 0.87–0.96). The AUC of the combined model was significantly greater than the AUC of the CSF model (p = 0.04) and the cognitive test model (p = 0.01). Therefore, the combination of cognitive tests and CSF analysis contributed significant added diagnostic value to using CSF biomarkers or cognitive tests separately, when predicting AD (Table 3, Fig. 1).

**Table 3:** Comparison of cognitive tests and CSF biomarkers (logistic regression analysis).

Dependentvariable	Type of independentvariables	Independent variablesin the model	OR (95% CI)	Correctlyclassified	AUC (95% CI)
MCI-AD compared with MCI-other dementias and stable MCI	Cognitive tests and CSF	MMSE (O & R)Aβ_42_<208TauClock drawing <4 p	0.64 (0.55–0.75)13.3 (3.90–45.2)1.02 (1.01–1.03)3.66 (1.19–11.3)	85%	0.93* (0.87–0.96)
	CSF	Aβ_42_TauAge	0.98 (0.97–0.99)1.02 (1.01–1.03)1.02 (1.00–1.05)	83%	0.89 (0.82–0.94)
	Cognitive tests	MMSE (O & R)AgeClock drawing <4 p	0.48 (0.37–0.63)1.10 (1.06–1.14)3.46 (1.28–9.31)	81%	0.85 (0.77–0.90)

* p<0.05 compared with AUC for cognitive tests and AUC for CSF. Note that some variable are continuous and others dichotomous, which greatly affects the OR.

The Hosmer and Lemeshow goodness-of-fit test was >0.05 for all models, indicating a good fit of the model to the data.

Demographic variables entered in all models: age and sex; CSF variables entered: Tau, Aβ_42_ or Aβ_42_ dichotomised at <208 and P-tau; Cognitive test variables entered: MMSE (orientation & recall) and clock drawing dichotomised at <4. All were entered with the backward LR method.

AUC  =  Area under the curve; CI  =  Confidence interval; MMSE (O & R)  =  the orientation and delayed word recall parts of the MMSE; MCI-AD  =  MCI patients who later convert to AD; MCI-other dementias  =  MCI patients who later convert to a dementia other than AD; OR  =  Odds ratio.

## Discussion

In this study, we found that there was no significant difference between the brief cognitive tests and the CSF biomarkers in predicting progression to AD. The combination of cognitive tests and CSF biomarkers was significantly better than both CSF biomarkers and cognitive tests used separately, thus providing a small added diagnostic value in predicting AD (Table 3, Fig. 1).

### The Cognitive Tests

In agreement with our results, previous studies have also found that the orientation and delayed recall parts of the MMSE are good at predicting specifically AD [15], [16], [28]. The reason MMSE (orientation & recall) can identify specifically MCI-AD among MCI-other dementias and stable MCI is that patients with subcortical dementias tend to produce relatively high scores on these parts of the MMSE, while AD deteriorate early in orientation and memory [35], [36].

Clock drawing has generally produced low predictive accuracies, consistent with our results, which have been significant in some studies [37], [38] but not in others [11], [14], [39]. It is possible that the clock drawing test would have performed better if a clock copying task had been used, since clock copying compared to clock drawing can discriminate between AD and subcortical dementias such as DLB and VaD [40]–[42].

### The CSF Biomarkers

A meta-analysis has investigated the ability of P-tau to separate stable MCI from MCI-AD. The pooled data from six studies indicated a sensitivity of 81% and a specificity of 65% [43], which are slightly lower predictive accuracies compared to the present study (sensitivity 67%, specificity 86%; Table 2). There is no meta-analysis for Aβ_42_ and Tau that examines progression of MCI, but Bloudek et al. pooled data from 15–24 studies to compare AD with other dementias and stable MCI. They found a sensitivity and specificity to be 73% and 72%, respectively, for Aβ_42_, and 77% and 74%, respectively, for tau. Both results are lower compared to the present study (Table 2). The better predictive accuracies in our study might be explained by the long follow-up period, which increases the likelihood of a correct diagnosis, and by the fact that the cut-offs were optimised for the present population.

### Logistic Regression Analyses

When comparing the AUCs of the CSF and cognitive tests models, no significant difference was found (p = 0.36). Thus, in this study the MMSE and the clock drawing test were about equally as good as the CSF biomarkers at identifying MCI-AD and differentiating it from MCI-other dementias and stable MCI.

When combining the cognitive tests and the CSF biomarkers, 85% of the cases were classified correctly and the AUC of 0.93 was significantly better than the cognitive test model (p = 0.01) and the CSF model (p = 0.04). Therefore, the combination of the MMSE, the clock drawing test and CSF biomarkers provided significant additional diagnostic value compared to using either method alone (Table 3; Fig. 1). Although statistically significant, it should be noted that the combined model only classified additionally 4% (5 patients) correctly compared to the cognitive tests and 2% (3 patients) compared to the CSF biomarkers.

In terms of clinical usage, CSF markers and cognitive tests should of course never be used alone to diagnose AD, but as a complement to strengthen the diagnosis. For this purpose, both instruments have been incorporated in the new clinical criteria for AD [5], [6].

### Comparison of Cognitive Tests and Biomarkers in Other Studies

To the best of our knowledge, only three previous papers have examined both CSF analysis and cognitive tests to predict the follow-up diagnoses of MCI [11], [18], [19]. In one of the papers, it was not investigated statistically if the combination provided significant added value for predicting AD [11]. The other two papers are from the Alzheimer’s Disease Neuroimaging Initiative (ADNI) study. In one of them, Llano et al. followed MCI patients during 12 months and found that the ADAS-cog (a 30–40 minutes long cognitive test battery) was equal to MRI of the brain and CSF analysis [19]. In the other, Ewers et al. found that the combination of different independent variables provided no significant added value [18]. In fact, they found that any one single variable was just as good as any combination of CSF, cognitive test and volumetric atrophy measurements. Although we had enough power in our study to detect a significant added value when combining cognitive tests and CSF biomarkers, the difference was quite small and our results corresponds well to that of Ewers et al. These findings suggest that roughly the same patients are identified regardless of the investigative method used.

### Methodological Remarks

An advantage of this study was the long follow-up period for patients with stable MCI (mean 5.9 years, range 3.2–8.8), which makes it the world’s second-longest follow-up study of CSF biomarkers in MCI patients [44]. Because most MCI patients convert to dementia within three years, this follow-up should suffice to ensure reliable diagnostic results [45]. The incidence of dementia was 13.8% and the incidence of AD was 10.1%, which is similar to that reported in other studies [46]. Unfortunately, we lacked some demographic data such as education, family history and mood disorders, which might have been of value to adjust for in the regression analyses.

The consensus group of physicians were blinded to the cognitive test results and CSF data from the initial visit, thus avoiding circular reasoning during the later analysis of the data. However, a possible confounder when evaluating cognitive tests as predictors of AD is that such tests give a measure of cognitive impairment, which is one of the criteria later used for the diagnosis. This means that the closer the MCI patients are to dementia (or to more obvious cognitive impairment), the better the cognitive tests should be in predicting conversion. If the patients were investigated, say, two years earlier, it is likely that the prediction accuracy of the tests would be worse whereas the CSF biomarkers would probably produce the same results.

The types of dementia diagnosed among the patients with MCI who developed dementia during follow-up correspond roughly to the prevalence of different dementia disorders in the community [47]–[50]. The result is also consistent with post-mortem studies, which have shown that a significant subset of patients with MCI exhibit neuropathological features associated with non-AD dementias [51]–[53]. To develop instruments that predict AD with high specificity in a clinical population, it is therefore important to include heterogeneous MCI populations, which include other prodromal dementias than AD.

### Conclusions

In this six-year follow-up study of MCI patients, we found that the MMSE and the clock drawing test were as accurate as the best combination of CSF biomarkers in identifying patients who will develop AD. This is the first study to compare these cognitive tests with CSF biomarkers, and it provides important information about the predictive value of brief cognitive tests, especially for those clinics in which CSF analysis is unavailable. The combination of CSF and cognitive tests showed significantly greater accuracy than CSF biomarkers or cognitive tests alone when predicting AD. The combination therefore provides a small added diagnostic value.
